# Cranberry Proanthocyanidins Mitigate Reflux-Induced Transporter Dysregulation in an Esophageal Adenocarcinoma Model

**DOI:** 10.3390/ph16121697

**Published:** 2023-12-07

**Authors:** Yun Zhang, Katherine M. Weh, Bridget A. Tripp, Jennifer L. Clarke, Connor L. Howard, Shruthi Sunilkumar, Amy B. Howell, Laura A. Kresty

**Affiliations:** 1 Section of Thoracic Surgery, Department of Surgery, University of Michigan, 1500 East Medical Center Drive, Ann Arbor, MI 48109, USA; yunzh@med.umich.edu (Y.Z.); kweh@med.umich.edu (K.M.W.); conhow@med.umich.edu (C.L.H.); sshruthi@umich.edu (S.S.); 2 Bioinformatics Core Research Facility, Center for Biotechnology, University of Nebraska—Lincoln, N300 Beadle Center, Lincoln, NE 68588, USA; bridget.tripp@huskers.unl.edu; 3 Department of Statistics and Department of Food Science Technology, Quantitative Life Sciences Initiative, University of Nebraska—Lincoln, 253 Food Innovation Center, Lincoln, NE 68583, USA; jclarke3@unl.edu; 4 Marucci Center for Blueberry and Cranberry Research, Rutgers University, 125A Lake Oswego Road, Chatsworth, NJ 08019, USA; ahowell@scarletmail.rutgers.edu

**Keywords:** cancer prevention, cranberry proanthocyanidins, plant polyphenols, reflux-induced esophageal adenocarcinoma, ATP-binding cassette transporters, solute carrier transporters, aquaporins, proton transporters, cation transporters, gastroesophageal reflux disease, Barrett’s esophagus

## Abstract

We recently reported that cranberry proanthocyanidins (C-PACs) inhibit esophageal adenocarcinoma (EAC) by 83% through reversing reflux-induced bacterial, inflammatory and immune-implicated proteins and genes as well as reducing esophageal bile acids, which drive EAC progression. This study investigated whether C-PACs’ mitigation of bile reflux-induced transporter dysregulation mechanistically contributes to EAC prevention. RNA was isolated from water-, C-PAC- and reflux-exposed rat esophagi with and without C-PAC treatment. Differential gene expression was determined by means of RNA sequencing and RT-PCR, followed by protein assessments. The literature, coupled with the publicly available Gene Expression Omnibus dataset GSE26886, was used to assess transporter expression levels in normal and EAC patient biopsies for translational relevance. Significant changes in ATP-binding cassette (ABC) transporters implicated in therapeutic resistance in humans (i.e., *Abcb1*, *Abcb4*, *Abcc1*, *Abcc3*, *Abcc4*, *Abcc6* and *Abcc10*) and the transport of drugs, xenobiotics, lipids, and bile were altered in the reflux model with C-PACs’ mitigating changes. Additionally, C-PACs restored reflux-induced changes in solute carrier (SLC), aquaporin, proton and cation transporters (i.e., *Slc2a1*, *Slc7a11*, *Slc9a1*, *Slco2a1* and *Atp6v0c*). This research supports the suggestion that transporters merit investigation not only for their roles in metabolism and therapeutic resistance, but as targets for cancer prevention and targeting preventive agents in combination with chemotherapeutics.

## 1. Introduction

The incidence of esophageal adenocarcinoma (EAC) has increased by more than 500% in the United States over the last 30 years, yet the 5-year survival rate remains less than 20% [1,2]. This is despite surgical and therapeutic advancements and the widespread use of esophagogastroduodenoscopy (EGD) and anti-reflux medications targeting Barrett’s esophagus (BE), the only recognized precursor lesion to EAC [3,4]. Persistent reflux of gastric and duodenal contents, known as gastroesophageal reflux disease (GERD), is the strongest known risk factor, long associated with BE and EAC progression [3,4,5,6,7,8,9]. Tobacco use is a stronger risk factor for esophageal squamous cell carcinoma (ESCC), but also imparts about 2-fold increased risk for EAC [10]. More recently, obesity has emerged as a strong consistent and dose-dependent risk factor contributing to BE and EAC [6,7,8,11,12,13,14]. BE patients are commonly treated with acid-suppressing medications such as proton pump inhibitors or H2 blockers; however, about half of these patients report incomplete responses to currently available treatments [15,16]. The standard of care for those diagnosed with EAC is currently chemotherapy and radiation, followed by surgery [17,18]. Unfortunately, less than 25% of EAC patients achieve a pathological complete response to treatment, as reflected by the dismal 5-year survival statistics [2,19]. Successful targeting of the known genomic alterations identified in EAC remains elusive. Thus, an improved understanding of understudied areas, such as transporters, may inform new adjuvant therapies or prevention strategies which are urgently needed to efficaciously target BE and EAC.

Our lab recently reported that cranberry proanthocyanidins (C-PACs) inhibit esophageal adenocarcinoma progression in the rat esophagogastroduodenal anastomosis (EGDA) model of reflux-induced EAC [20]. In this model, the duodenum is anastomosed to the gastroesophageal junction, leading to the reflux of bile and acidified gastric contents into the esophagus [21], mimicking GERD in humans. C-PACs inhibited esophageal EAC by 83% through reversing reflux-induced bacterial, inflammatory, and immune-implicated proteins and genes, with concomitant reductions in esophageal bile acids which drive EAC progression [20]. Herein, we extend these findings to (1) investigate whether C-PACs’ mitigation of bile reflux-induced transporter dysregulation may mechanistically contribute to EAC inhibition in the rat reflux-induced EAC model and (2) characterize our preclinical results in the context of transporter dysregulation reported in human EAC, BE or normal squamous esophageal samples from non-cancer patients to inform the translational relevance of our findings.

Transporters govern the active or passive movement of a broad range of metabolites and compounds including ions, peptides, small molecules, lipids, and macromolecules across biological membranes. There are three main classes of membrane transporters which include ATP-binding cassette (ABC), P-type ATPases, and the solute carrier (SLC) family [22,23,24,25]. Transport may also occur via proton and cation transporters or in the case of water or glycerol through aquaporins [26,27]. Transporters mediate the influx or efflux of a variety of compounds, with some acting in a bidirectional manner, including many SLC transporters. The SLC superfamily includes over 450 transport proteins across 65 families, based on sequence similarity [22,28]. SLC transporters are involved in the influx or bidirectional movement of small molecules including glucose (SLC2) [29,30], bile acids (SLC6, SLC10, SLC31, SLC27, SLC51, SLCO) [31,32,33], various cations (SLC8, SLC9, SLC22, SLC24, SLC44, SLC47) [34,35,36,37], bicarbonate (SLC4) [38], and amino acids (SLC1, SLC3, SLC6, SLC7, SLC36, SLC38, SLC43) [39,40,41,42,43,44]. The transport of glutamine, a non-essential neutral amino acid, is conducted by SLC families 1, 6, 7 and 38, and the intracellular requirements of this amino acid are necessary for the synthesis of the antioxidant glutathione [40]. With respect to chemotherapeutic treatment of EAC patients, a number of SLC family members, including SLC22A2, are responsible for the uptake of 5-fluorouracil (5-FU), cisplatin, carboplatin, and paclitaxel, and in turn impact cancer cell death, pathological progression, therapeutic efficacy, and patient prognosis [45,46,47,48,49,50,51,52,53,54,55]. ABC transporters are primary active transporters that utilize energy from ATP hydrolysis to move a wide range of substrates generally to the outside of a cell [22]. ABC substrates are varied, including lipids, sterols, bile acids, ions, small molecules, large polypeptides, and multiple chemotherapeutic agents and other xenobiotics [25]. Additionally, members of this transporter superfamily include ABCB2 (TAP1) and ABCB3 (TAP2) transporters, which are associated with MHC class I peptides involved in immune responses, multi-drug resistance, and the identification of microbial pathogens through the recognition of pathogen-associated microbial patterns (PAMPs) [56,57,58]. The overexpression of select ABC transporters can result in a more rapid efflux of therapeutic agents from the cell reducing contact time and treatment efficacy [24,25]. Thus, ABC transporters are known to play a critical role in the development of multidrug resistance in numerous cancers, including EAC [50,51,59,60]. Most studies evaluating transporters in the context of esophageal cancer have focused on ESCC and the role of transporters in therapeutic resistance, with fewer studies characterizing transporters linked to EAC. To date, studies have reported transporter dysregulation correlated with progression to EAC, therapeutic resistance or poor patient prognosis [43,46,47,50,51,53,54,55,61,62,63,64,65,66]. Moreover, *ABCB1* was recently identified as a driver gene of EAC supporting a role beyond drug resistance [61].

Aquaporin transporters are multimeric channel proteins composed of two subfamilies that transport either water alone or water and small molecules including glycerol and urea [67]. Members in the latter subfamily are known as aquaglyceroporins and include *AQP3*, AQP7, *AQP9* and *AQP10* [26]. Increased levels of *AQP1*, *AQP3* and *AQP5* have been reported in ESCC with *AQP1* and *AQP5* expression levels negatively correlated with patient survival [68,69,70]. In cancer cells, aquaporins facilitate cell migration, cell division, cell adhesion, and the tissue water balance [71].

There is increasing recognition that transporter genes are essential for many cellular processes and that gene or protein level changes in transporters can cause or contribute to human cancer at multiple stages, not just therapeutic resistance. To date, publications documenting the effects of cranberry proanthocyanidins on transporters are lacking. There is a single report of cranberry juice modulating the levels of P-glycoprotein (P-gp), or MDR1, encoded by *ABCB1*, a major efflux transporter of xenobiotics with a well-documented role in therapeutic resistance [61]. Cranberry juice decreased the levels of P-gp in rat enterocytes and increased levels in rat hepatocytes [72]. Cranberry juice administered to mice reportedly caused drug interactions with OATP (*SLCO*) substrates as well [73]. Other plant-based polyphenols have been reported to modulate the gene expression of *ABCB1*, altering P-gp levels and impacting drug bioavailability and the response of cancer cells to chemotherapeutic treatment [74,75,76,77]. Limited preclinical work also supports a role for cranberry extracts in increasing cisplatin sensitivity in ovarian cells, but without a linkage to transport mechanisms [78]. Overall, the role of transporters in cancer initiation and progression remains understudied, and in turn, whether transporters may be viable targets for cancer prevention is unclear. The current study sheds new light on C-PACs’ significant impact on multiple transporters in a preclinical model for reflux-induced EAC and provides translational context through comparisons with the published literature and a human EAC dataset that had not previously been mined for transporter changes [48].

## 2. Results

### 2.1. C-PACs Mitigate Reflux-Induced Alterations in ABC Transporter Expression in the Rat Esophagus

To characterize transporter expression, we performed RNA sequencing and RT-PCR on esophageal RNA isolated from water-, C-PAC- and reflux-exposed rat esophagi with and without C-PAC treatment in the drinking water, as previously detailed [20]. Specifically, we utilized the Bio-Rad PrimePCR transporters plate, which is a predesigned assay containing optimized primers for 87 unique transporters and reference genes. Table 1 shows the ABC transporters that are significantly altered in reflux-induced EAC animals and mediated by C-PAC treatment.

Fourteen ABC transporters were significantly altered in reflux-induced EAC, including 12 efflux and 2 influx ABC transporters. The cholesterol and lipid transporter *Abca9* was the only ABC transporter significantly downregulated in reflux-induced EAC. Conversely, reflux upregulated 13 of 14, or 92.9%, of the altered ABC transporters. Reflux induced the bile salt transporters *Abcb1*, *Abcb11*, *Abcc3* and *Abcc5*, the lipids and fatty acid transporters *Abca1* and *Abca9* and transporters associated with chemotherapeutic drug resistance, including multiple Abcc family members, as well as *Abcb1b* and *Abcg2*. Among the reflux-induced ABC transporters, *Abcb11* showed the largest magnitude of induction with a log2 fold-change of 3.28. *Abcb11* encodes the bile salt export pump (BSEP) and is known to transport bile acids and play a role bile acid metabolic process [79]. C-PACs non-significantly reduced *Abcb11* (log2FC: −1.99, *p* = 0.128). Overall, C-PAC treatment resulted in the potent mitigation of reflux-induced ABC transporter dysregulation, as evidenced by the significant reversal of 64.3% (9/14) of reflux-induced transporter alterations.

### 2.2. C-PACs Mitigate Reflux-Induced Alterations in SLC Transporter Expression in the Rat Esophagus

Thirty SLC transporters belonging to twenty different families were significantly altered in reflux-induced EAC (Table 2), including fourteen influx transporters and sixteen bidirectional transporters. Twenty-four transporters (24/30, or 80%) were significantly upregulated in reflux-induced EAC, including the glucose transporters *Slc2a1* and *Slc5a1*, the amino acid transporters *Slc3a2*, *Slc7a5*, *Slc7a7*, *Slc7a8* and *Slc7a11*, the lactate transporters *Slc16a2*, *Slco2a1*, *Slco4a1* and proton pumps such as *Slc4a11*, *Slc9a1*, *Slc9a3*, *Slc9a5* and *Slc15a2*. Several SLC transporters of bile were also induced by reflux, including *Slc6a14*, *Slc6a20*, *Slc10a2* and *Slc31a1*. Six transporters (6/30 or 20%) were significantly downregulated in reflux-induced EAC, including metal ion transporters (*Slc4a9*, *Slc8a3*, and *Slc24a3*), the peptide transporter *Slc15a2*, the organic cation and cisplatin transporter *Slc22a2* and the oxoglutarate and glutathione transporter *Slc25a11*. C-PAC treatment significantly (*p* ≤ 0.05) mitigated reflux-induced dysregulation in 76.7% (23/30) of the individual SLC transporters identified and 80% at the family level. Additional transporters dysregulated by reflux were mitigated by C-PACs based on directionality, but did not reach statistical significance, as shown in the tables.

### 2.3. Aquaporin and Additional Transporters Dysregulated in Reflux-Induced EAC and Restored by C-PACs

In addition to transcriptional level changes in ABC and SLC family transporters, alterations in vacuolar-type ATPase, aquaporins, major vault proteins (MVPs), and voltage-dependent anion channels (VDACs) were also assessed (Table 3), considering their roles in immune modulation and the acidification of cellular compartments supporting cancer progression and development [26,80,81,82]. Reflux-induced EAC significantly upregulated the expression of two vacuolar-type ATPases, *Atp6v0a4* and *Atp6v0c*, with C-PAC administration significantly restoring the expression of both ATPases (Table 3). Three aquaporins (*Aqp1*, *Aqp3*, and *Aqp4*) were significantly dysregulated by reflux, with C-PACs non-significantly mitigating the expression of aquaporins. In addition, the upregulation of *Mvp* and *Vdac2* was also observed in reflux-induced EAC, with C-PACs significantly modulating the expression of *Mvp*.

### 2.4. Transporter Expression Altered by C-PACs in the Normal Rat Esophagus

Among all transporters reported to be mitigated by C-PACs in reflux-induced EAC above, the significant downregulation of four transporters, including *Abcb1b*, *Abcb3*, *Slc22a8* and *Atp6v0c*, was also observed in the normal rat esophagus treated with C-PACs compared to water-treated controls (Table 4). C-PACs not only modulate reflux-induced transporter changes but also impact select transporters in the normal non-reflux exposed esophagus.

### 2.5. Transporter Dysregulation Observed in Human Esophageal Cancer and Corresponding Pathway Enrichment

To better understand the translational relevance of transporter dysregulation identified in the reflux-induced EAC model and C-PAC mitigation, we analyzed the publicly available GEO dataset GSE26886, as referenced in Table 5. GSE26886 included data from 19 normal esophageal samples isolated from non-cancer patients and data from 21 EAC patients [48]. Nineteen transporters dysregulated in the rat reflux-induced EAC model were also found to be dysregulated in the human GEO dataset and, to our knowledge, reported herein for the first time (Table 5). In addition, we also reviewed other published studies and identified transporters altered in the rat reflux-induced EAC model in common with alterations identified in previously published studies in human EAC, BE, and normal esophageal tissues, or human BE or EAC cell lines (Table 6). Esophageal squamous cell carcinoma (ESCC)-related studies were reviewed, but given differences in the etiology, molecular drivers, metabolic dysregulation and therapeutic responsiveness, we focused on EAC-related studies [83,84,85,86].

Overall, 58.8% (30/51) of transporters altered in the rat reflux-induced EAC model were altered in human EAC (Table 5 and Table 6). Among the 14 ABC transporters dysregulated in the reflux-induced EAC animal model, 11 (78.6%) were also found to be dysregulated in human EAC, whereas 56.7% (17/30) of the SLC transporters altered in the reflux model were also identified as altered in human EAC studies. Upregulation of *Aqp1* and *Mvp* was also found in human studies, further supporting the suggestion that dysregulation of transporters observed in the rat reflux-induced EAC model is consistent with many of the transporters dysregulated in human EAC.

**Figure 1:**
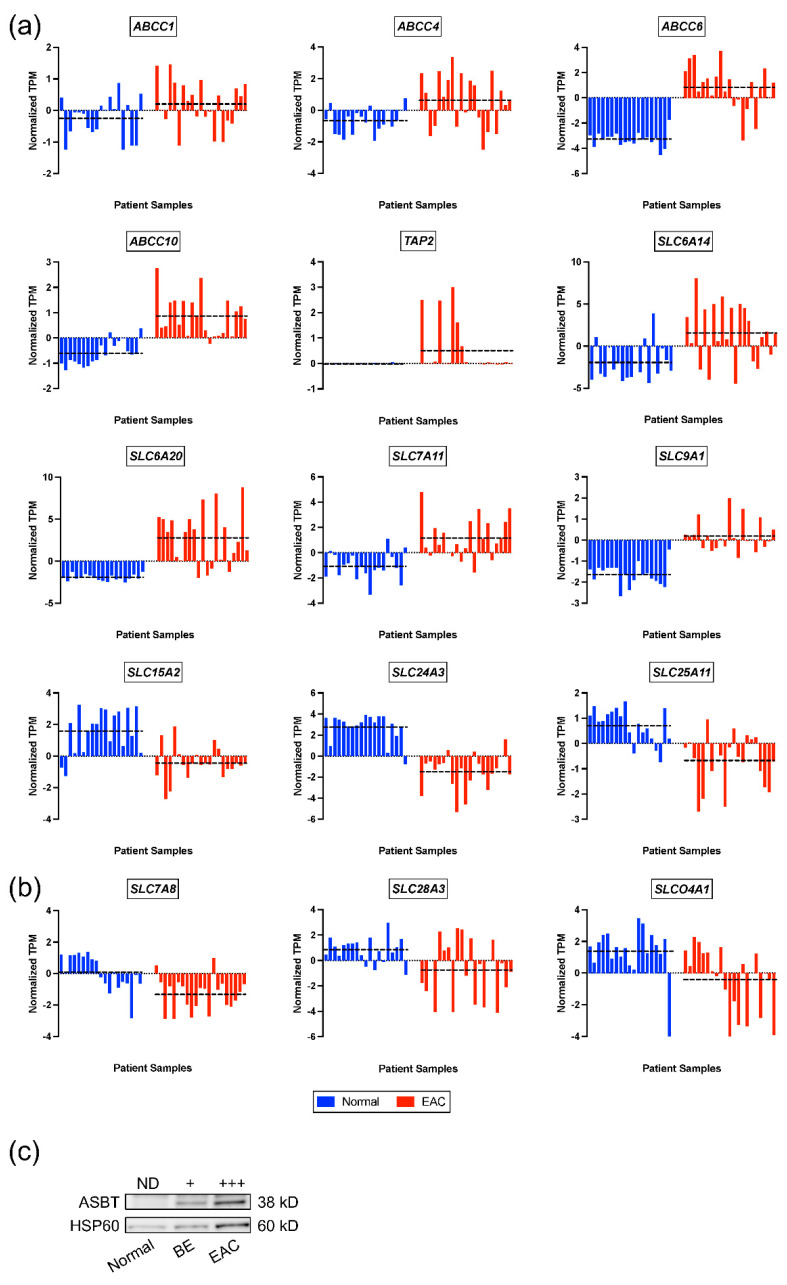
Dysregulation of ABC and SLC transporters in human EAC. (**a**) Differentially expressed transporters identified in the human EAC GEO26886 dataset comparing normal non-cancer tissues to EAC patient tissues and with the same directionality of change observed in the reflux-induced EAC rat model. (**b**) Differentially expressed transporters in the human EAC GEO26886 dataset with the opposite directionality compared to the rat reflux-induced EAC model. Dashed lines indicate the mean normalized transcript-per-million (TPM) value in each group. (**c**) Immunoblot of ASBT (encoded by *SLC10A2*) in patient-matched normal, BE, and EAC samples. ND, not detected.

Next, individual expression values of multiple transporters with changes in alignment between the rat reflux-induced EAC model and human EAC cases were plotted in Figure 1a, showing expression differences in transporters when comparing normal esophageal tissue from non-cancer patients to EAC tissue (GEO26886) [48]. A small number of transporters dysregulated in the rat reflux-induced EAC model were modified in the opposite direction in the human dataset, as displayed in Figure 1b. Opposing changes seemed to be driven by patient heterogeneity in either normal esophageal (*SLC7A8*) or EAC tissues (*SLC28A3* and *SLCO4A1*). As proof-of-concept, immunoblotting was performed on ASBT, an SLC transporter encoded by *SLC10A2* (Table 2), using lysates isolated from human EAC, BE, and matched distant normal esophageal tissue (Figure 1c). The Western blot results showed the upregulation of ASBT in both BE and EAC tissue samples compared with normal esophageal tissue levels, which is in alignment with mRNA results in both the reflux-induced EAC animal model and human EAC tissue, as compared to normal esophageal tissues.

**Figure 2:**
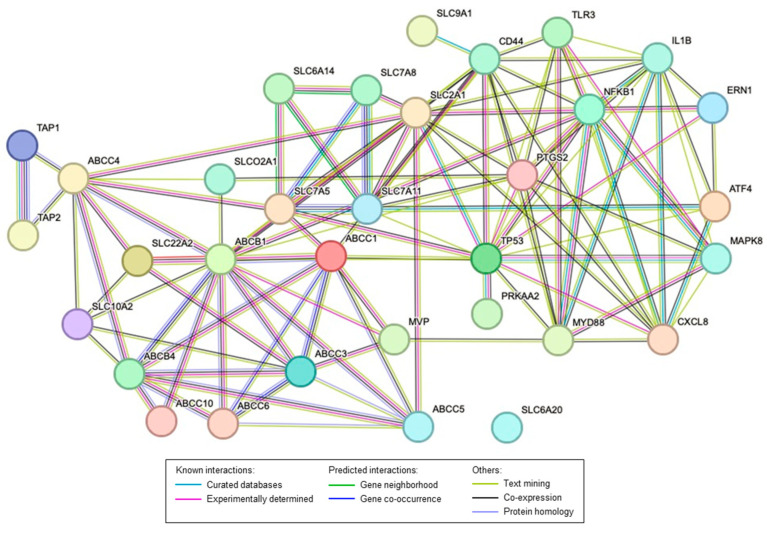
STRING network interaction of the major transporters and proteins altered in the rat reflux-induced EAC model and mitigated by C-PACs. Colored lines as shown in the legend define the basis and the type of interaction between the molecules in the model. All transporters included have been identified as being altered in human EAC compared to normal esophageal tissues from non-cancer patients.

**Figure 3:**
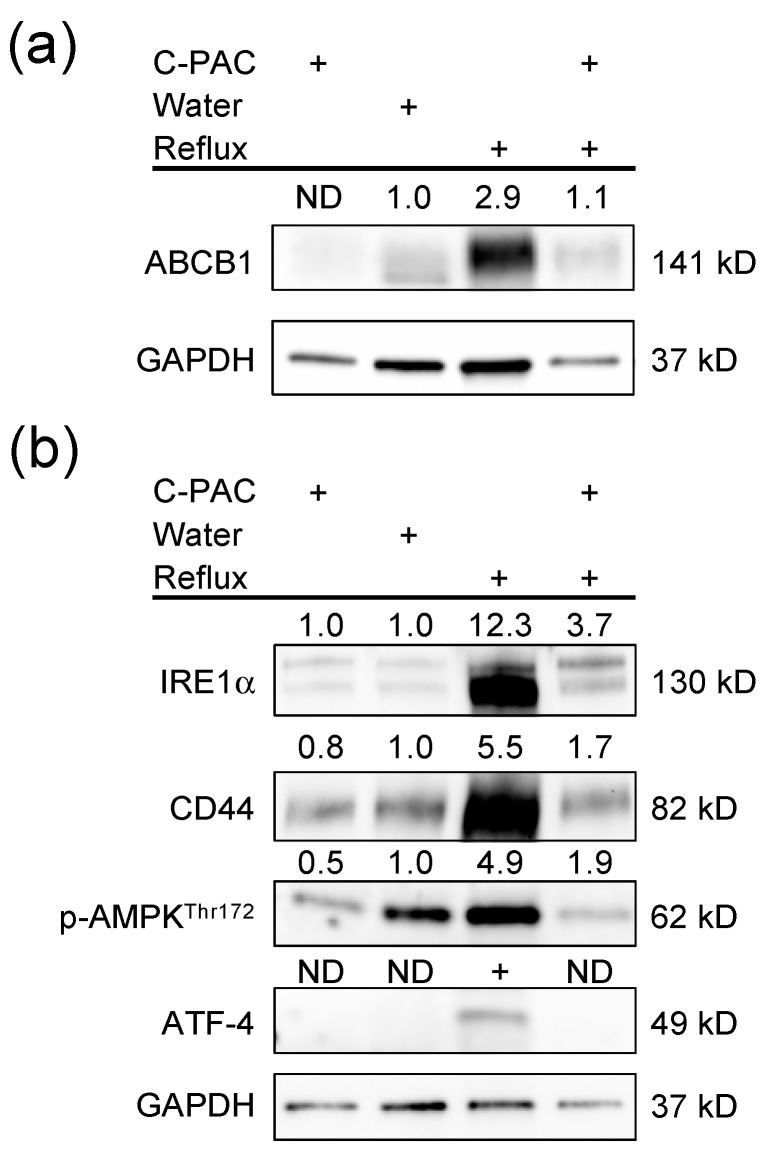
C-PACs mitigate the dysregulation of transporters and key regulatory genes in reflux-induced EAC. (**a**) Dysregulation of ABCB1 in reflux-induced EAC and mitigation by C-PACs. (**b**) Reflux-induced EAC led to increased expression of stress response-related proteins and upregulation of CD44 with linkages to inflammation, bacterial sensing and the immune state. C-PACs mitigate reflux-induced protein induction. ND, not detected.

A STRING protein interaction prediction was performed (Figure 2) using a list of 20 key transporters that are dysregulated in the same direction in both animal and human data, along with key proteins altered in the rat reflux-induced EAC model and mitigated by C-PACs, as previously described and evaluated herein (Figure 3) [20]. The protein interaction prediction revealed a complex connection within transporters and between other key proteins dysregulated or mutated in EAC. Notably, TP53, the most commonly mutated gene in EAC [94], has direct protein interactions with several transporters including ABCC1, SLC2A1 and SLC7A5. CD44, a commonly upregulated protein in EAC [95], also has direct protein interactions with ABCB1, ABCC1, SLC2A1, SLC7A11 and SLC9A1. All transporters, except for SLC6A20, have either direct or indirect interactions with key regulatory proteins previously reported in EAC, including the transcription factors ATF4 and NFκB, unfolded protein response-related proteins (ERN1 or IRE1, ATF4) and multiple inflammation, bacterial and immune-linked proteins (i.e., PTGS2/Cox2, MYD88, IL-8 or CXCL8, IL-1β, CD44) [96,97,98,99]. The string network further illustrates the connectivity between families of transporters (ABCs and SLCs).

Enrichment analysis of the human EAC dataset revealed GO processes significantly dysregulated in EAC tissues compared to normal esophageal tissues with top changes linked with the transporters reported above (Table 1, Table 2, Table 3 and Table S1), including primary metabolic processes (*ABCC6*, *SLC25A11* and *SLC25A13*), organic substance metabolic processes (*ABCC5*, *ABCC6*, *ABCC10*, *SLC9A1*, *SLC25A11* and *SLC25A13*), and cellular responses to stress (*SLC7A11*, *SLC7A5* and *SLC9A1*). Although there is limited availability of validated transporter antibodies with rat specificity, we did perform a Western blot with anti-ABCB1 and other transporter-linked proteins. Increased ABCB1 expression was observed in the reflux-induced rat EAC model, with C-PACs significantly mitigating ABCB1 expression (Figure 3a). A panel of immunoblots of interacting proteins of transporters was performed (Figure 2 and Figure 3b). Reflux-induced EAC led to the increased expression of stress response-related proteins, such as phospho-AMPK, IRE1α, ATF-4, as well as CD44, and C-PACs strongly reversed the reflux-induced expression levels.

## 3. Discussion

Transporters are integral membrane proteins essential to the uptake, distribution, metabolism and excretion of exogenous compounds and endogenous metabolites, including xenobiotics, nutrients, hormones, bile acids, peptides, lipids, sugars and drugs [22,23,24,25,26,100]. Most transporters are members of either the SLC or ABC superfamilies, with a smaller fraction categorized as aquaporins or ATPases. Transporters act as gatekeepers of cellular contents and as such have critical roles in a broad range of cellular and physiological processes which impact normal cellular functions, disease pathologies and response to drug therapies [22]. It is well established that the overexpression of select ABC transporters leads to multidrug resistance (MDR), contributing to chemotherapy failure and poor patient prognosis [101]. As an example, ABCB1, or MDR1 (also known as P-glycoprotein), has been extensively studied in part due to its broad substrate specificities and linkages to therapeutic resistance in cancers, including limited reports in EAC [63,88]. Transporters not only influence absorption and excretion, but also distribution and metabolism. Considering that altered metabolism is recognized as a cancer hallmark, targeting transporters may not only potentiate therapeutic efficacy but also enhance cancer prevention efforts. However, the comprehensive characterization of transporters in the target and tumor of interest is needed to better understand metabolic vulnerabilities and in turn select agents that effectively mitigate transporter dysregulation in a context-specific manner. Beyond drugs, many natural cancer inhibitors, including polyphenols, reportedly modulate transporter expression and may offer a better safety profile [74,75,102,103,104].

We recently reported that C-PACs, rich in cranberry polyphenols, significantly inhibited EAC in a rat model through mitigating reflux-induced bacterial, inflammatory and immune-implicated proteins and genes as well as reducing esophageal bile acids, but through unknown processes [20]. These observations combined with metabolic enrichment results supported the hypothesis that C-PACs potentiate reflux-induced changes in bacterial metabolites, amino acids, fatty acids, bile acids and the TCA cycle, raising the question of transporter involvement [20]. In this study, we first comprehensively characterized bile reflux-induced transporter alterations in a rat EAC model and investigated C-PACs’ capacity to mitigate reflux-induced transporter dysregulation. Second, preclinical study results were compared to transporter level changes in human studies through a search of the published literature and through reanalyzing a publicly available dataset in which we compared transporter mRNA expression levels in normal esophageal tissues from non-cancer patients to the levels in EAC tissues. The GEO dataset utilized for comparison purposes was unique in that few datasets include expression data from normal esophageal tissues from non-cancer patients.

Our research findings identified a total of 51 transporters significantly altered at the mRNA level upon reflux induction in the rat EAC model, including ABC (*n* = 14), SLC (*n* = 30), and ATPase and aquaporin (*n* = 7) family members. Herein we report for the first time that C-PACs significantly reversed reflux-induced changes in 69% of the individually identified transporters. At the family level, C-PACs reversed 75%, 80%, and 33% of the ABC, SLC, and AQP/Atpases/voltage transporters, respectively. These data support the hypothesis that C-PACs have potent effects in mitigating reflux-induced esophageal transporter changes. Next, to understand the translational relevance of these results, we compared our preclinical model findings to the literature and the human expression dataset described above. Fifty-eight percent of individual transporters (30/51) dysregulated in the rat reflux model were significantly altered in human EAC or precursor lesions. Moreover, we identified a number (*n* = 12; Table 5) of transporters differentially expressed in human EAC compared to normal esophageal tissues that had not previously been reported, including *ABCC1*, *ABCC6*, *SLC6A14*, *SLC6A20*, *SLC7A7*, *SLC7A11*, *SLC15A2*, *SLC24A3*, *SLC25A11*, *SLC25A13*, *AQP1* and *MVP*. These transporters have documented roles in immunity (SLC15A2, AQP3), transporting proton pump inhibitors (ABCB1), chemotherapeutic resistance (ABCB1), cell cycle transition (ABCB1), glutathione conjugation or metabolic processes (ABCC6, SLC7A11, SLC25A11), prostaglandin transport (ABCC6), the transport of bile (SLC6A14, SLC6A20), NRF2 signaling (SLC6A14, SLC6A20, SCL7A11), L-arginine transport and metabolism (SLC7A7, SLC6A14), cystine and L-glutamate transport (SLC7A11), the TCA cycle (SLC6A14), the oxidative stress response, ferroptosis and the p53 transcriptional gene network (SLC7A11), calcium homeostasis and the regulation of gene expression (SLC24A3), gluconeogenesis (SLC25A11, SLC25A13), the response to hypoxia, the regulation of retinoic acid, vitamin D and keratinocyte differentiation (AQP3) and ERBB and EGFR signaling (MVP), among others.

Of those transporters previously identified in EAC studies (Table 6) and altered in the rat reflux model (*n* = 18), the majority were reported in the context of histopathological progression to EAC (*n* = 9) [46,50,53,54,62,64,90,92], followed by linkages to therapeutic resistance (*n* = 4) [51,55,61,66] and patient prognosis or survival (*n* = 4) [45,62,63,65]. Substrates for these transporters consist of glucose, glutamine, glycine, tryptophan, omeprazole, Hh2 blockers, bile, chemotherapeutic and other drugs, cholesterol, lipids, leukotrienes, glutathione, sodium, calcium, bicarbonate and polyphenols, among others. In turn, these transporters have documented roles in many cancer-related processes including the response to bacterium or LPS (SLC10A2, ABCB1), antigen presentation (ABCB2, ABCB3), the regulation of cellular pH (SLC9A family members), bile secretion or response to bile or bile metabolism or transport (ABCA1, ABCB1, ABCB4, ABCC3, ABCC4, ABCC5, ABCC10), NRF2 signaling (ABCC3, SLC6A20), xenobiotic metabolism (ABCA1, ABCB1, ABCC3, ABCC4, ABCC5), cell migration (SLC9A1), cell communication and hypoxia (SLC8A3), leukocyte migration (SLC7A8), glycolysis and gluconeogenesis (SLC2A1) and the TCA cycle (SLC7A5/8, SLC25A11). Reprogramming of glucose metabolism via glycolysis or the Warburg effect is thought to provide cancer cells with an energetic advantage for growth, metastasis, and immune escape; however, emerging evidence supports the suggestion that some tumors favor oxidative metabolism and the TCA cycle over aerobic glycolysis [105,106]. Recent research evaluating the metabolic and immune differences between ESCC and EAC reported that EAC relies more on oxidative metabolism, the catabolism of glycolipids, the electron transport system and TCA cycle activation [86]. In alignment, previous research reported a shift from glycolytic to oxidative metabolism under acidic conditions [107]. Similarly, we noted TCA cycle enrichment in the rat reflux-induced EAC model with significant mitigation by C-PACs [20]. Herein we identified several transporters with roles in cellular respiration, glycolysis, gluconeogenesis and the TCA cycle, as evidenced by the transport of glucose, fatty acids, glutamine and oxoglutarate. There has been strong interest in the development of drugs to target these metabolic pathways in recent years. However, to date, the success of treating cancers with glucose metabolism modifier drugs has been limited due to unacceptable toxicities, short half-life and solubility issues [108]. To better understand the specificity and potency of C-PACs, future studies should include comparative analysis with other known drugs or transporter modulating agents, particularly those approved for testing in human cohorts.

We also noted a few transporters which were moving in the opposite direction in the human dataset compared to the rat reflux model, including SLC28A3 and SCLO4A1, which show strong heterogeneity in the EAC tumor samples, potentially contributing to the opposing trend. Genetic variants in transporters may also contribute to the heterogeneous expression of select transporters at the mRNA level in individual patients [109]. Additionally, post-translational modifications are important for the structure, function and regulation of transporters [110]. To date, over 400 modification types have been identified, with phosphorylation, glycosylation, and ubiquitination events being among the most common modifications [110,111,112,113,114,115,116,117]. As a result, mRNA- and protein-based results may not align, as documented for SLC7A5, a transporter of glutamine and various xenobiotics identified herein. Thus, research in characterizing specific transporter alterations at the genomic level in conjunction with the transcript and protein levels would be highly informative.

Overall, our results show the rat reflux-induced EAC model shares patterns of transporter dysregulation with those identified in EAC patients compared to non-cancer controls or as reportedly linked to EAC progression, therapeutic resistance or poor patient prognosis [45,46,50,51,53,54,55,61,62,63,64,65,66,90,92]. Importantly, C-PACs showed strong capacity to mitigate reflux-induced transporter dysregulation in the rat EAC model when delivered at a concentration that is behaviorally achievable as part of the normal diet [20]. Bioactive concentrations of C-PACs can be reached by consuming 2–4 ounces of 100% cranberry juice, 8–10 ounces of a 27% cranberry juice cocktail, and about a 1/4 cup of fresh cranberries or a 1/3 cup of sweetened dried cranberries, each providing 60 to 80 mg of C-PACs. To our knowledge, C-PACs have not previously been investigated for their effects on transporter mitigation. However, these results are in alignment with limited preclinical research evaluating the effects of cranberry juice on transporters. Cranberry juice was previously found to modulate the levels of P-gp, a protein encoded by the major MDR efflux transporter ABCB1 [72]. The latter study also illustrated target-specific effects reporting decreased levels of P-gp in rat enterocytes and increased levels in rat hepatocytes [72]. Our results also align with the larger body of literature reporting P-gp modulation by a host of other polyphenols including but not limited to quercetin, kaempferol, tea catechins, epigallocatechin, curcumin, honokiol, magnolol and resveratrol [74,75,76,77]. Moreover, C-PACs not only potentiated reflux-induced transporter changes in reflux-exposed esophageal samples but also impacted select transporters in the normal non-reflux exposed esophagus, suggesting that C-PACs may modify transporters in the healthy esophageal epithelium, potentially serving a protective function. C-PACs significantly reduced the levels of *Abcb1* in both the normal and reflux-induced esophagus. This transporter exhibits important roles in the context of EAC, BE and GERD. It is responsible for the efflux of omeprazole, commonly prescribed for the management of GERD [118], has a documented role in xenobiotic detoxification and chemotherapeutic resistance, and recently was identified as a driver of EAC [61]. *Slc22A8*, with a role in the response to toxic substances and processing xenobiotics, was similarly downregulated, which aligns with previous research by our group showing that C-PACs mitigate bile-induced reductions in the detoxification enzyme GSTT2 in normal patient-derived primary cell lines [119]. The *Atp6v0c* transporter, with roles in maintaining pH homeostasis and cellular acidification, was also downregulated by C-PACs in the normal rat esophagus, as well as the reflux-exposed esophagus. A shift in the acid–base balance promotes cancer cell proliferation, apoptosis resistance, invasiveness, metastasis, immune evasion and therapeutic resistance [120]. ATP6V0C was recently reported to enhance aerobic glycolysis and cell motility utilizing in vitro models for ESCC, whereas the depletion of this transporter, commonly dysregulated in ESCC, attenuated cancer-associated cell proliferation and invasion and suppressed glucose metabolism through interactions with pyruvate kinase isoform M2, a key glycolysis regulator [121]. In the normal esophagus, C-PACs also significantly suppressed *Abcb3* (TAP2), a transporter involved in multi-drug resistance, T-cell-mediated cytotoxicity, antigen presentation and response to bacterial pathogens. This transporter was upregulated in the rat reflux-induced esophagus, as was TAP1. Similar to results in the rat reflux-induced EAC model, we identified both TAP1 and TAP2 to be induced in human EAC compared to normal tissues, and others have reported high TAP1 and TAP2 expression to be significantly associated with poor overall survival among EAC patients [65]. C-PACs significantly mitigated the levels of a number of transporters in the rat reflux model which have been linked to poor patient prognosis in EAC patients, including ABCB4, ABCC4, and SLC2A1. Each of these transporters has documented roles in chemotherapeutic resistance. Additionally, SLC2A1 is a major facilitator glucose transporter overexpressed in numerous cancers, including EAC, and with roles not only in glycolysis but also epithelial–mesenchymal transition, hypoxia, cell-cycle regulation and DNA repair [122].

Taken together, these data show that the dysregulation of transporters occurs in both the rat reflux-induced EAC model and reflux-driven EAC in humans. Importantly, C-PACs significantly mitigate reflux-induced transporter dysregulation in the rat model of EAC with concomitant inhibition of cancer progression [20]. Relevantly, the majority of transporters modified in the rat reflux model have documented roles in histopathological progression to EAC, therapeutic resistance, or survival among EAC patients, illustrating the translational potential of these findings. Beyond transport, C-PACs exert prebiotic activity abrogating reflux-induced dysbiosis and mitigating reflux-induced bile acid metabolism and immune modulation, culminating in the inhibition of EAC through TLR/NF-κB/TP53 signaling [20]. In alignment, many of the transporters dysregulated in the current study have roles in immune regulation, the response to bacteria, detoxification, the processing of xenobiotics, regulating cellular pH and the transport of bile acids, known to drive BE progression to EAC.

The totality of evidence supports the involvement of transporters across a broad range of metabolic and cancer-associated processes impacting the full cancer continuum from initiation to promotion, progression and metastasis, as well as treatment resistance and patient outcomes. Thus, research targeting transporters should include cancer prevention interventions as well as those focused on enhancing therapeutic efficacy. Research is currently underway to investigate whether C-PACs act synergistically with standard-of-care chemotherapeutics to induce EAC cell death via mechanisms involving transporters, as an example. Non-toxic agents with bioactivity when consumed at levels achievable in the normal diet, like C-PACs, are especially promising for targeting transport mechanisms to inhibit cancer or improve therapeutic efficacy. However, much research remains to be completed to address whether C-PACs act as true transporter substrates or simply as modifying agents. Additional research is warranted focusing on high-priority transporters as identified herein. We are employing genetic and pharmacological targeted approaches coupled with phenotypic readouts to further investigate the role of C-PACs compared to other agents in targeting transporters. Lines of research should include substrate uptake assays, binding assays and fluorescent-based functional assays designed to detect changes in membrane potential, intracellular pH and localization. Ultimately, unraveling the roles of esophageal transporters may inform new targets for cancer prevention and treatment interventions. In closing, because the characterization of transporters in the esophagus is limited, particularly in the context of EAC or BE precursor lesions, future studies in larger cohorts with defined genomics as well as transcriptional and protein level results would prove informative for future targeting efforts with transporter modulating agents.

## 4. Materials and Methods

### 4.1. Esophagogastroduodenal Anastomosis (EGDA) Surgical Model of Reflux-Induced EAC and C-PAC Delivery

EAC was induced in male Sprague Dawley rats using the EGDA surgical reflux model, as previously described [20]. Briefly, one week after surgery, animals were randomized to receive either water or C-PACs (690 μg/rat/day) in the drinking water ad libitum. C-PACs were prepared and characterized as previously described [123]. Rats were sacrificed at 40 weeks of study and esophageal tissue flash frozen in liquid nitrogen and stored at −80 °C until processing for downstream analysis.

### 4.2. Rat Esophageal RNA Isolation, RNA Sequencing and Transporter Expression Analyses

RNA was isolated from rat lower esophageal tissue using the RNeasy Fibrous Tissue Kit (Qiagen, Germantown, MD, USA). Each sample was homogenized in 400 µL of Buffer RLT with beta-mercaptoethanol for 3 (10 s) pulses with a handheld homogenizer (Pro-Scientific Inc., Oxford, CT, USA). RNA was purified following the manufacturer’s instructions and eluted in 20 µL of Ambion RNA Storage Solution (Thermo Fisher Scientific, Waltham, MA, USA). RNA concentration and quality were measured using the RNA 6000 Pico kit on the Bioanalyzer 2100 capillary electrophoresis system (Agilent, Santa Clara, CA, USA) and stored at −80 °C. Isolated RNA was utilized for both RNA sequencing and targeted transporter plate evaluation via qRT-PCR. Four micrograms of RNA per sample was reverse transcribed using the iScript™ Advanced cDNA Synthesis Kit (Bio-Rad, Hercules, CA, USA) using the following protocol: priming at 25 °C for 5 min, reverse transcription at 46 °C for 20 min and RT inactivation at 95 °C for 1 min. Expression levels of 87 genes were assessed via the PrimePCR Drug Transporters (SAB Target List) R384 rat plate (Catalog #10047102, Bio-Rad) using 1X SsoAdvanced Universal SYBR Green Supermix (Bio-Rad). Real-time PCR was performed on the CFX384 real-time PCR system (Bio-Rad) using the following protocol: activation at 95 °C for 2 min, followed by 40 cycles of denaturation at 95 °C for 5 s and annealing/elongation at 60 °C for 30 s. Data were analyzed using CFX Manager (Bio-Rad) where relative changes in gene expression were calculated using 2^−ΔΔCt^, where ΔΔCt = ΔCt (Reflux) − ΔCt (Water) or ΔΔCt = ΔCt (Reflux + C-PAC) − ΔCt (Reflux) and Log_2_ transformed. Data were normalized to the expression levels of Gapdh and Hsp90ab1 and four to six animals were assessed per treatment group. RNA sequencing (RNA-seq) of the isolated RNA, as described above, was performed by BGI Americas (San Jose, CA, USA) with 100 bp paired-end reads using the Illumina HiSeq 4000 sequencing platform. RNA-seq analysis, including adapter trimming, reads mapping, and differential gene expression analysis, was performed using Qiagen CLC Genomics Workbench (version 20.0.4, https://digitalinsights.qiagen.com, accessed 4 November 2020) with default parameters. mRatBN7.2 was used as the reference genome. Transporters included in the data tables were chosen based on statistical significance (*p* ≤ 0.05) in the reflux-induced group.

### 4.3. GEO Dataset Renormalization and Analysis

The previously published NCBI GEO dataset GSE26886 was utilized to assess transporter dysregulation in human esophageal tissue [48]. GSE26886 contains gene expression profiling of 19 normal esophageal squamous epithelium samples and 21 EAC samples originally published by Wang et al. on the Affymetrix Human Genome U133 Plus 2.0 array. CEL files were downloaded and renormalized using the gcrma package (version 2.0) in R (version 3.6.2, R Core Team; www.r-project.org, accessed 12 March 2020) to determine differentially expressed genes in normal versus EAC samples in GSE26886 [124]. Log2FC was calculated for each marker using the following equation: Log2FC = Log2(EAC) − Log2(Normal).

### 4.4. Tissue Lysate Isolation and Western Blot Analysis

Frozen rat esophagus tissue was homogenized in Tissue Protein Extraction Reagent (ThermoFisher, Waltham, MA, USA) using TissueLyser II (Qiagen) at 30 Hz for 5 min. Extracted protein was then quantified using the DC Protein Assay (Bio-Rad). Western blot analysis was performed as previously described [123]. Images were captured using the Bio-Rad ChemiDoc Imaging System and quantified by means of chemiluminescent immunodetection using Bio-Rad Image Lab Software version 6.1.0 with expression levels normalized to the loading control GAPDH. Immunoblotting was performed using commercially available antibodies from Abcam (Cambridge, MA, USA) and Cell Signaling Technology (Danvers, MA, USA): ABST (ab203205, 1:500), ATF-4 (CST #11815, 1:1000), CD44 (ab189524, 1:500), GAPDH (CST #2118, 1:25,000), HSP60 (CST #12165, 1:5000), IRE1α (CST #3294, 1:1000), and phospho-AMPK (CST #2535, 1:1000). Patient EAC samples with matched normal and BE tissues were collected at the University Hospital at the University of Michigan. Informed consent was obtained from patients prior to sample collection. Protein extraction and quantification were similarly performed as described above.

### 4.5. Pathway Analysis and Protein Interaction Prediction

Pathway analysis was performed using the list of significantly differentially expressed genes (*p*-value and FDR ≤ 0.05) using Metacore and Cortellis Solution software (https://clarivate.com/products/metacore/, accessed 25 October 2023, Clarivate Analytics, London, UK). Enrichment analysis was used to identify enriched pathways. Protein interaction prediction between transporters and key dysregulated proteins in EAC was performed using the STRING database (version 12.0, https://string-db.org, accessed 25 October 2023) with default parameters [125].

### 4.6. Statistical Analyses

Statistical analyses were performed in GraphPad Prism software (version 10.0.3, GraphPad Software, Boston, MA, USA, www.graphpad.com, accessed 20 October 2023). A Student’s *t*-test was applied for pairwise comparisons of gene expression data. For GSE26886, significance between normal and EAC samples was determined in MATLAB software version 9.3.0 (Natick, MA, USA) using a one-sided Student’s *t*-test with Bonferroni–Hochberg FDR correction for multiple comparisons. *p*-values ≤ 0.05 and FDR ≤ 0.05 were considered statistically significant.

## 5. Conclusions

This is the first study to report C-PAC’s capacity to mitigate transporter dysregulation in the context of EAC prevention. Our study showed that low-dose aqueous delivery of C-PAC in a rat EAC model significantly modulated expression of cancer-associated transporters involved in immune regulation, response to bacteria, detoxification of xeonobiotics, bile acid transport, glycolysis, TCA cycle, histopathological progression and therapeutic drug resistance. The translational relevance of our preclinical findings was confirmed through comparing to the body of human transporter literature and reanalysis of a human EAC data set which revealed a strong overlap of transporter changes in human and rat EAC. Transporter alterations detected in this study align logically with our findings that C-PAC inhibits EAC by abrogating reflux-induced gut dysbiosis and esophageal bile acid metabolism through TLR/Nf-κB/TP53 signaling. Our results support that transporters are essential in multiple key cellular processes linked to EAC causation and progression, as well as therapeutic resistance. In turn, non-toxic agents like C-PAC warrant further evaluation to assess whether the positive preclinical findings are efficacious in human cohorts at risk for EAC progression or whether C-PAC may act synergistically with chemotherapeutics to enhance treatment efficacy.

## Figures and Tables

**Table 1:** Esophageal reflux-induced ABC transporter expression and modulation by C-PACs.

Gene	Protein/ Alias	Substrate(s); Function(s) in Addition to Transport	Influx or Efflux	Reflux-Induced Changes		C-PAC-Induced Changes Given Reflux	
				Log2FC ^a^	*p*-Value ^b^	Log2FC ^a^	*p*-Value ^b^
*Abca1*	ABC1	Cholesterol, phospholipids, bile salts; Cellular response to cholesterol, LPS, retinoic acid, cytokine and xenobiotic stimulus	Efflux	1.21	0.037	−0.58	0.498
*Abca9*	ABCA9	Lipids, cholesterol, acyl CoA derivatives; Lipid homeostasis, monocyte differentiation	Efflux	−2.20	0.002	0.73	0.018
*Abcb1b*	MDR1 P-gp	Chemotherapeutics, cholesterol, phospholipids, bile salts, omeprazole, statins, antibiotics, immunosuppressants antivirals; G2/M transition of mitotic cell cycle, xenobiotic detoxification, xenobiotic metabolic process	Efflux	1.80	0.001	−2.65	0.002
*Abcb2*	TAP1	Peptides or antigens; Adaptive immune response, antigen processing and presentation via MHC class I, defense response	Influx	1.18	0.048	−1.34	0.295
*Abcb3*	TAP2	Peptides or antigens; T cell mediated cytotoxicity, antigen processing and presentation via MHC class I, response to molecule of bacterial origin	Influx	1.96	0.019	−1.71	0.188
*Abcb4*	MDR3	Phospholipids, paclitaxel, bile salts; Bile acid secretion, cellular response to bile, lipid homeostasis	Efflux	0.65	0.007	−1.31	0.048
*Abcb11*	BSEP	Bile acids, phospholipids, statins; Bile acid metabolic process, bile acid signaling pathway, cholesterol, lipid and phospholipid homeostasis	Efflux	3.28	0.021	−1.99	0.128
*Abcc1*	MRP1	Glutathione, leukotriene C4, estradiol-17-beta-o-glucuronide, methotrexate, chemotherapeutic drugs, xenobiotics; Cell chemotaxis, anti-cancer drug resistance, cellular response to amyloid-beta, oxidative stress, leukotriene metabolic process, heme and xenobiotic catabolic process	Efflux	1.19	0.011	−1.28	0.027
*Abcc3*	MRP3	Bile acids, etoposide, leukotriene C4, glucuronisides, xenobiotics; Xenobiotic metabolic process, metabolism of lipids, steroids and bile, recycling of bile, nuclear receptors meta-pathway, NRF2	Efflux	1.54	0.001	−1.47	0.042
*Abcc4*	MRP4	cAMP, cGMP, cholate, statins, GSH, bile salts, prostaglandin, urate; Xenobiotic metabolism; cellular detoxification, anti-cancer drug resistance	Efflux	1.54	0.001	−2.06	<0.001
*Abcc5*	MRP5	cAMP, cGMP, folate, glutathione, glutamate, heme, bile salts, antiretroviral nucleosides, thiopurine anticancer drugs; Hyaluronan biosynthetic process, xenobiotic metabolic process	Efflux	1.31	0.034	−1.51	0.054
*Abcc6*	MRP6	Glutathione conjugates, ATP, cisplatin, leukotriene C4; Calcium homeostasis, gene expression, inhibition of non-skeletal tissue mineralization, response to xenobiotic stimulus	Efflux	1.06	0.041	−2.31	0.041
*Abcc10*	MRP7	Chemotherapeutics, cholesterol, bile, phospholipids, leukotriene C4, glutathione, peptides; Leukotriene metabolic process; heme synthesis; cellular detoxification	Efflux	0.15	0.040	−1.77	0.003
*Abcg2*	BCRP	Xenobiotics, urate, lipids, riboflavin, doxorubicin, estradiol, imatinib, irinotecan, statins, tamoxifen, testosterone; Cellular detoxification, urate metabolic process	Efflux	1.74	0.014	−1.54	0.212

Gene expression changes by treatment were determined based on ^a^ PrimePCR Drug Transporter pathway plate results and ^b^ *p*-values determined by Student’s *t*-test; ABC: ATP-binding cassette transporter; C-PACs: cranberry proanthocyanidins.

**Table 2:** Esophageal reflux-induced SLC transporter expression and modulation by C-PACs.

Gene	Protein/ Alias	Substrate(s); Function(s) in Addition to Transport	Influx or Efflux	Reflux-Induced Changes		C-PAC-Induced Changes Given Reflux	
				Log2FC	*p*-Value ^c^	Log2FC	*p*-Value ^c^
*Slc2a1* ^a^	GLUT1	Glucose, galactose, mannose, glucosamine, ranitidine, quercetin, resveratrol; Glycolysis, gluconeogenesis, cellular respiration	Influx	1.98	0.008	−1.93	0.044
*Slc3a2* ^a^	CD98	Large neutral amino acids; Calcium regulation, lymphocyte activation	Both	1.65	0.005	−1.66	0.128
*Slc4a9* ^b^	AE4	Sodium, chloride, bicarbonate; Anion exchange, Intracellular pH	Both	−2.23	<0.001	1.98	<0.001
*Slc4a11* ^b^	BTR1	Borate, sodium, bicarbonate, protons; Cell proliferation, response to oxidative stress	Both	2.37	0.029	−2.52	0.004
*Slc5a1* ^a^	SGLT1	Glucose, galactose, myo-inositol, sodium; Nuclear receptors meta-pathway, NRF2	Influx	1.23	0.046	−1.06	0.392
*Slc6a14* ^b^	ATB0+	Neutral and cationic amino acids, glutamine, arginine, glycine, bile salts, metal ions, amines, sodium and chloride neurotransmitters; Response to toxic substance, nuclear receptors meta-pathway, NRF2	Both	7.53	<0.001	−2.16	<0.001
*Slc6a20* ^b^	SIT1	Sodium, chloride, amino acids, proline, bile salts, amines, sarcosine, pipecolic acid; Nuclear receptors meta-pathway, NRF2, kidney function	Both	3.50	<0.001	−1.43	0.004
*Slc7a5* ^a^	LAT1	Large neutral amino acids, xenobiotics; Aryl hydrocarbon receptor pathway, nuclear receptors meta-pathway, response to LPS, autophagy regulation	Both	1.40	0.004	−1.59	0.017
*Slc7a7* ^a^	y + LAT	Cationic amino acids, large neutral amino acids; Regulation of arginine metabolic process	Both	1.92	0.028	−1.32	0.266
*Slc7a8* ^a^	LAT2	Cationic amino acids, large neutral amino acids, glycine, proline, tryptophan, thyroid hormone, toxins; Leukocyte migration, metal ion homeostasis	Both	1.93	0.001	−2.10	0.007
*Slc7a11* ^a^	xCT	Cystine, L-glutamate; Oxidative stress response, glutathione metabolic process, nuclear receptors meta-pathway, NRF2, ferroptosis, p53 transcriptional gene network	Both	2.51	0.001	−2.53	0.003
*Slc8a3* ^b^	NCX3	Sodium, calcium; Cell communication, cellular response to hypoxia, memory	Both	−3.19	<0.001	2.13	0.008
*Slc9a1* ^b^	NHE1	Protons, sodium, hydrogen; Cellular pH, cell migration, cell volume; Response to hypoxia, response to acidic pH, cell polarity and migration, RhoA, p38, and ErbB1 signaling	Both	1.00	<0.001	−0.84	<0.001
*Slc9a3* ^b^	NHE3	Protons, sodium, hydrogen; Regulation of intracellular pH	Both	3.12	0.002	−1.01	0.830
*Slc9a5* ^b^	NHE5	Protons, sodium, hydrogen; Regulation of intracellular pH	Both	1.93	<0.001	−1.45	<0.001
*Slc10a2* ^b^	ASBT	Bile salts, sodium, phospholipids; Cholesterol homeostasis, response to bacterium	Influx	2.37	0.003	−1.42	0.047
*Slc15a2* ^a^	PEPT2	Di- and tri- peptides, protons, beta-lactam antibiotics, xenobiotics; Innate immune response, xenobiotic detoxification	Influx	−2.09	0.050	0.42	0.087
*Slc16a2* ^a^	MCT8	Thyroid hormones (T2, rT3, T3, T4), lactate; Amino acid and thyroid hormone metabolic process	Influx	3.08	<0.001	−3.11	0.004
*Slc22a2* ^a^	OCT2	Organic cations, oxaliplatin, cisplatin, carboplatin, paclitaxel, 5-fluorouracil, ranitidine, metformin; Kidney function	Influx	−2.64	0.026	0.92	0.038
*Slc22a7* ^a^	OAT2	Organic anions, acyclovir, prostaglandins, xenobiotics; Fluoropyrimidine activity and pathway, xenobiotic metabolism	Influx	1.57	0.023	−2.97	0.018
*Slc22a8* ^a^	OAT3	Organic anions, carboxylate, prostaglandins, xenobiotics; Response to toxic substances	Influx	2.09	0.032	−2.89	0.015
*Slc24a3* ^b^	NCKX3	Calcium, sodium, potassium; Bone mineralization, calcium homeostasis, regulation of gene expression	Influx	−1.67	<0.001	0.85	0.021
*Slc25a11* ^b^	OGC	Oxoglutarate, malate, glutathione; Gluconeogenesis from lactate; Nitrogen metabolism, apoptosis	Both	−1.17	<0.001	1.00	<0.001
*Slc25a13* ^a^	CTLN2	Aspartate, glutamate; ATP biosynthetic process, cellular respiration, gluconeogenesis, response to calcium	Both	1.40	0.024	−1.14	0.254
*Slc28a3* ^a^	CNT3	Nucleosides, vitamins, utidine, gemcitabine, fludarabine, ribavirin; Xenobiotic metabolic process	Influx	0.89	0.021	−1.95	0.024
*Slc31a1* ^a^	CTR1	Copper, cisplatin, bile salts, organic acids, metal ions, amines; Copper homeostasis and metabolism, platinum pathway, angiogenesis	Influx	1.47	0.003	−1.39	0.137
*Slc35f2* ^b^	HSNOV1	Amino acids, glucose, nucleotides, lipids, organic anions; Biological process	Influx	1.50	<0.001	−1.05	0.001
*Slc46a2* ^b^	TSCOT	Cyclic GMP-AMP; T cell homeostasis, innate immune response, regulation of T cell differentiation	Influx	1.32	<0.001	−0.77	0.015
*Slco2a1* ^a^	OATP2A1	Prostaglandins, lactate, vitamins, nucleosides; Lipid metabolism in senescent cells	Both	1.67	0.011	−1.64	0.037
*Slco4a1* ^a^	OATP4A1	Thyroid hormones (T3, T4, rT3), esterone-3-sulfate, organic anion, taurocholate; Intracellular pH, regulation of pH	Influx	1.10	0.024	−2.84	0.026

Gene expression changes by treatment were determined based on ^a^ PrimePCR Drug Transporter pathway plate or ^b^ RNA-sequencing data with ^c^ *p*-values determined by Student’s *t*-test; SLC: solute carrier transporter; C-PACs: cranberry proanthocyanidins.

**Table 3:** Esophageal reflux-induced expression of aquaporin and other transporters and modulation by C-PACs.

Gene	Protein/ Alias	Substrate(s); Function(s) in Addition to Transport	Influx or Efflux	Reflux-Induced Changes		C-PAC-Induced Changes Given Reflux	
				Log2FC	*p*-Value ^c^	Log2FC	*p*-Value ^c^
*Atp6v0a4* ^b^	Subunit of ATPase	Protons for ATP hydrolysis; Intracellular pH reduction, regulation of pH	Influx	2.73	<0.001	−2.34	<0.001
*Atp6v0c* ^b^	Subunit of ATPase	Protons for ATP hydrolysis; Acidification, intracellular pH reduction, regulation of macroautophagy and Wnt signaling pathway	Influx	1.73	0.005	−1.91	0.006
*Aqp1* ^a^	AQP1	Water, ammonium, carbon dioxide, glycerol, nitric oxide; cGMP-mediated signaling, cell volume homeostasis, cellular response to UV, cAMP, copper ion, hypoxia, nitric oxide, retinoic acid, Gram-negative bacterium	Both	0.11	0.018	−1.01	0.089
*Aqp3* ^b^	AQP3	Water, glycerol; Cellular response to hypoxia, retinoic acid and vitamin D, regulation of keratinocyte differentiation and immune system process	Both	2.40	0.008	−1.62	0.289
*Aqp4* ^b^	AQP4	Water; Cellular response to type II interferon, intracellular water homeostasis,	Both	−2.72	<0.001	2.10	0.200
*Mvp* ^b^	MVP	Nucleo-cytoplasmic transport, mRNA; ERBB signaling pathway, regulation of: EGFR signaling pathway, protein tyrosine kinase activity, protein autophosphorylation	Efflux	1.00	0.003	−0.77	0.012
*Vdac2* ^a^	VDAC2	Closed: cation-selective; Open: weak anion selectivity; Negative regulation of intrinsic apoptosis signaling pathway	Both	1.27	0.016	−0.83	0.483

Gene expression changes by treatment were determined based on ^a^ PrimePCR Drug Transporter pathway plate or ^b^ RNA-sequencing results with ^c^ *p*-values determined by Student’s *t*-test; SLC: solute carrier transporter; C-PACs: cranberry proanthocyanidins.

**Table 4:** C-PACs alter transporter expression in the normal rat esophagus.

Gene	C-PACs versus Water	
	Log2FC	*p*-Value ^a^
*Abcb1b*	−1.75	0.037
*Abcb3*	−1.48	0.015
*Slc22a8*	−1.99	0.016
*Atp6v0c*	−1.78	0.018

^a^ *p*-values determined by Student’s *t*-test.

**Table 5:** Transporters significantly dysregulated in the human EAC dataset (GSE26886) in parallel with transporters altered in the rat reflux-induced EAC model (*p* < 0.05).

Gene Symbol	Log2FC	*p*-Value	FDR
*ABCB2*	1.71	7.14 × 10^−6^	5.92 × 10^−5^
*ABCB3*	0.50	3.90 × 10^−2^	1.04 × 10^−1^
*ABCB4*	0.96	9.77 × 10^−3^	3.37 × 10^−2^
*ABCC1*	0.46	4.38 × 10^−2^	1.14 × 10^−1^
*ABCC3*	3.92	1.77 × 10^−15^	1.80 × 10^−13^
*ABCC4*	1.29	3.56 × 10^−3^	1.43 × 10^−2^
*ABCC6*	2.18	8.78 × 10^−5^	5.55 × 10^−4^
*ABCC10*	1.49	1.52 × 10^−8^	2.40 × 10^−7^
*SLC6A14*	3.53	4.80 × 10^−4^	2.50 × 10^−3^
*SLC6A20*	4.72	1.35 × 10^−7^	1.69 × 10^−6^
*SLC7A7*	3.64	4.95 × 10^−13^	2.50 × 10^−11^
*SLC7A11*	1.52	1.22 × 10^−3^	5.64 × 10^−3^
*SLC9A1*	1.83	2.31 × 10^−11^	7.32 × 10^−10^
*SLC15A2*	−2.04	5.43 × 10^−6^	4.63 × 10^−5^
*SLC24A3*	−3.87	1.43 × 10^−11^	4.79 × 10^−10^
*SLC25A11*	−1.39	1.12 × 10^−5^	8.89 × 10^−5^
*SLC25A13*	1.92	1.49 × 10^−5^	1.15 × 10^−4^
*AQP1*	1.77	2.18 × 10^−3^	9.39 × 10^−3^
*MVP*	1.47	1.45 × 10^−7^	1.80 ×10^−6^

FDR determined using the Benjamini and Hochberg procedure.

**Table 6:** Transporters altered in the rat reflux-induced EAC model in parallel with identified changes previously reported in human esophageal adenocarcinoma or precursor lesions.

**Transporter**	**Target Tissues** **or Cell Lines**	**References**	**Summary Findings**
*ABCB1*	EAC, GEJ	[55,59,61,62,63,87,88]	Novel driver of EAC; Inc levels (39% in EAC) based on DNA copy number changes; gain reported in EAC; amplification in GEJ tumors compared to gastric samples; elevated expression in EAC compared with normal tissues and higher in EAC compared with SCC; negatively correlated with IC50 for 5-FU in human cell lines; chemotherapeutic resistance
*ABCB2*	EAC	[65]	Increased expression level in EAC, linked to reduced survival, and immune response; high mRNA expression level significantly linked to poor survival among EAC patients
*ABCB3*	EAC	[65]	High mRNA expression level significantly linked to poor survival among EAC patients
*ABCB4*	EAC, EC	[62,63]	Amplification in GEJ tumors compared to gastric samples; gain associated with poor survival
*ABCC3*	EAC, BE	[50,51,66]	Increased mRNA expression level from esophageal squamous epithelium to BE and with progression to EAC; increased mRNA expression in EAC cell lines linked to 5-FU resistance; SNP associated with response to platinum-based neoadjuvant therapy in EC patients (EAC and ESCC cases combined)
*ABCC4*	EAC	[62]	Gain in EAC and linked to poor survival
*ABCC5*	EAC	[89]	Associated with BE progression to EAC
*ABCC10*	EAC, GEJ	[64]	Amplification in 18% of EAC and GEJ tumors
*ABCG2*	EAC	[60,61]	Enhancer element in untranslated region identified as a noncoding driver element in EAC; increased mRNA expression in the OE19 EAC cell line following 5-FU treatment
*SLC2A1*	EAC	[45,46,47]	Associated with EAC and poor prognosis; increased expression in EAC compared with dysplasia’s; increased expression in EAC and high-grade dysplasias compared with non-dysplasias
*SLC7A5*	EAC	[54]	Increased mRNA expression and decreased LAT1 at the protein level in EAC compared with BE
*SLC7A8*	EC	[51]	Identified as part of a five-gene signature identifying SNPs impacting the response of esophageal cancer patients (combined ESCC and EAC) to platinum-based neoadjuvant therapy
*SLC8A3*	EAC	[90]	Mutated in a Chinese cohort of EAC patients and enriched in the protein digestion and absorption pathway (directionality not reported)
*SLC9A1*	EAC	[52,91,92]	DCA treatment increased levels in BE and EAC human derived cell lines; increased by bile exposure in the BE cell line; increased in BE patient samples and a dysplastic BE cell line
*SLC9A3*	EAC, BE	[53]	Identified as a potential novel risk loci among associated variants through the integration of expression quantitative trait loci and genetic association data in BE/EAC tissues
*SLC10A2*	EAC	[50]	Increased mRNA expression in BE and EAC compared to normal squamous esophagus
*SLC22A2*	EAC	[55]	Expression level impacted sensitivity to 5-FU treatment based on EAC human cell line treatment
*SLCO2A1*	Reflux-exposed	[93]	Increased expression in patients with reflux extending to the proximal esophagus, concomitant infiltration of CD3-positive lymphocytes and reduction in proximal esophageal TEER, increased in IL-8 and IL-1β and decreased occludin mRNA levels

## Data Availability

Data is contained within the article and Supplementary Material.

## Supplementary Materials

The following supporting information can be downloaded at: https://www.mdpi.com/article/10.3390/ph16121697/s1, Table S1: Top GO processes significantly dysregulated in EAC tissues compared to normal esophageal tissues from non-cancer patients.
