# Supplementary material for: Cranberry Proanthocyanidins Mitigate Reflux-Induced Transporter Dysregulation in an Esophageal Adenocarcinoma Model
Source: Pharmaceuticals (Basel). 2023 Dec 7;16(12):1697. doi: 10.3390/ph16121697 (PMC10747310; doi:10.3390/ph16121697)
Supplement: Supplementary file 1 [file pharmaceuticals-16-01697-s001.zip › pharmaceuticals-2746966-supplementary.pdf]

Table S1. Top Go processes significantly dysregulated in EAC tissues compared to normal esophageal tissues from non-cancer patients.

**GO Processes (Top 35/283)**	**Total**	**In Data**	***P*-value**	**FDR**
Primary metabolic process	5035	3006	3.202E-18	4.476E-14
Nitrogen compound metabolic process	4711	2814	2.076E-16	1.451E-12
Metabolic process	5728	3367	3.890E-15	1.813E-11
Organic substance metabolic process	5457	3205	2.302E-13	8.046E-10
Macromolecule metabolic process	4301	2557	1.448E-12	4.050E-09
Macromolecule modification	1937	1209	3.243E-12	7.556E-09
Protein modification process	1852	1154	2.394E-11	4.782E-08
Nucleobase-containing compound metabolic process	2191	1345	1.336E-10	2.334E-07
Cellular metabolic process	4361	2572	2.438E-10	3.787E-07
Cellular nitrogen compound metabolic process	2585	1568	3.396E-10	4.455E-07
Regulation of nitrogen compound metabolic process	4131	2442	3.505E-10	4.455E-07
Heterocycle metabolic process	2338	1425	5.386E-10	6.275E-07
Organelle organization	2195	1341	9.463E-10	1.018E-06
Regulation of primary metabolic process	4281	2521	1.228E-09	1.226E-06
RNA metabolic process	1355	852	1.700E-09	1.584E-06
Cellular response to stress	1506	940	1.887E-09	1.649E-06
Cell cycle	1025	655	4.588E-09	3.773E-06
Regulation of cellular metabolic process	4097	2410	8.962E-09	6.961E-06
Nucleic acid metabolic process	1780	1093	1.358E-08	9.991E-06
Regulation of cell cycle	1090	689	2.474E-08	1.730E-05
Organonitrogen compound metabolic process	3499	2069	3.183E-08	2.119E-05
Cellular aromatic compound metabolic process	2398	1443	5.287E-08	3.360E-05
Protein metabolic process	2737	1634	8.731E-08	5.308E-05
Regulation of metabolic process	4901	2846	1.363E-07	7.943E-05
Positive regulation of nitrogen compound metabolic process	2762	1646	1.488E-07	8.322E-05
Epithelium development	1168	729	1.886E-07	9.897E-05
Mitotic cell cycle	649	422	1.997E-07	9.897E-05
Renal system development	395	268	2.029E-07	9.897E-05
Tube development	1095	686	2.053E-07	9.897E-05
Regulation of macromolecule biosynthetic process	2848	1690	4.541E-07	2.116E-04
